# Di­chlorido­(4-methyl­aniline-κ*N*)[*N*-(4-methyl­phen­yl)-1-(thio­phen-2-yl)methanimine-κ*N*]palladium(II)

**DOI:** 10.1107/S2056989022004960

**Published:** 2022-05-17

**Authors:** Ray J. Butcher, Puspendra Singh, Gulam Shabbani

**Affiliations:** a Department of Chemistry, Howard University, 525 College Street NW, Washington, DC 20059, USA; b Department of Chemistry, Dr. Shakuntala Misra National Rehabilitation University, Lucknow, Uttar Pradesh 226017, India; University of Neuchâtel, Switzerland

**Keywords:** crystal structure, palladium mono-amine complex, *p*-toluidine, fingerprint plots

## Abstract

The structure of a mono-amine Pd^II^ complex, [PdCl_2_(C_7_H_9_N)(C_12_H_11_NS)], which crystallizes in the triclinic space group, *P*  , is reported. The primary geometry around the Pd^II^ atom closely resembles square planar (τ_4_′ = 0.069). In the (*E*)-1-(thio­phen-2-yl)-*N*-(*p*-tol­yl)methanimine ligand, the phenyl and thio­phene rings are not coplanar, subtending a dihedral angle of 38.5 (1)° because of steric effects. The PdCl_2_N_2_ coordination plane is almost perpendicular to the planes of the coordinated *o*-toluidine and the NC_2_ fragment [dihedral angles of 84.7 (1) and 72.50 (4)°, respectively]. The Pd—NH_2_ length of 2.040 (2) Å is slightly shorter than the observed mean value for other complexes involving a Pd atom attached to the nitro­gen of an aniline derivative. The mol­ecules display an inter­esting supra­molecular synthon based on reciprocal inter­molecular N–H⋯Cl hydrogen-bonding inter­actions of the *p*-toluidine amine fragment, which results in centrosymmetric dimeric units. These units are further linked by C—H⋯Cl inter­actions, resulting in chains in the *c*-axis direction where the mean-planes of the repeating fragment are oriented in the (110) plane.

## Abstract 2

The structure of a mono-amine Pd^II^ complex is reported in which the Pd—NH_2_ length is slightly shorter than the observed mean value for other complexes involving Pd attached to the nitro­gen of an aniline derivative.

## 1. Chemical context

The chemistry of monodentate mono-amine Pd^II^ compounds with amine ligands is of inter­est because the hydrogen bond between the amine and the catalyst plays a key role in the catalytic transformation of simple, easily accessible amines into highly substituted, biologically important amine-containing mol­ecules and pharmaceutical agents (Calleja *et al.*, 2015 ▸). While mono-amine Pd^II^ complexes are generally unstable and are formed as inter­mediates during the reaction, the corresponding bis­(amine) Pd^II^ complex is stable and ultimately hampers the utility of these compounds in the C—H activation reaction. Probably because of this, well-characterized mono-amine Pd^II^ complexes are relatively rare. In this article we report a well-characterized and room-temperature-stable mono-amine Pd^II^ complex. 
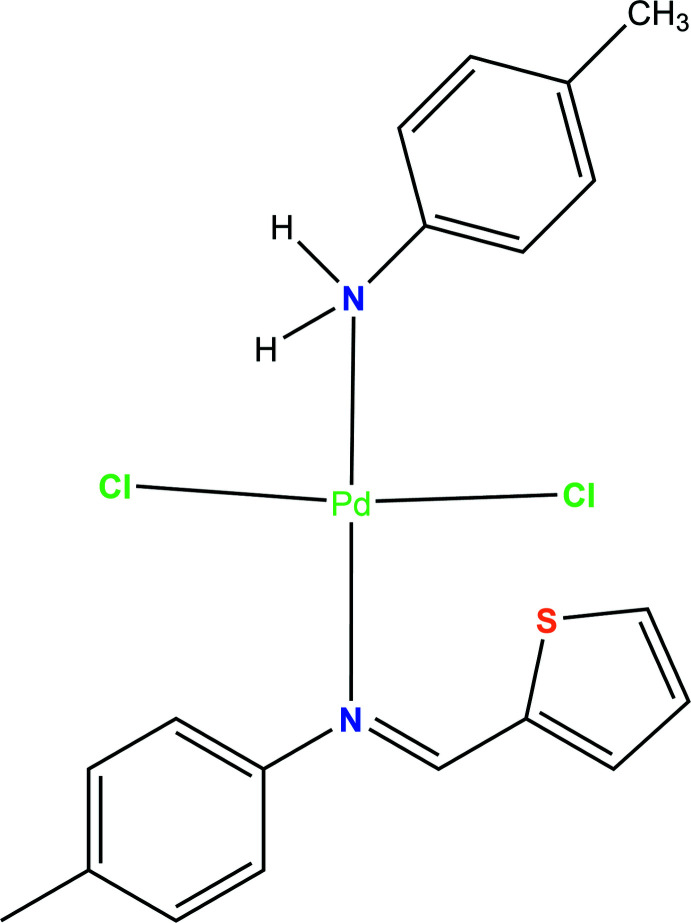


## 2. Structural commentary

In the Schiff base ligand H*L*1 used [H*L* ^1^ = (*E*)-1-(thio­phen-2-yl)-*N*-(*p*-tol­yl)methanimine and *L* ^2^ = *p*-toluidine], we expected that the *ortho* proton of the tolyl ring of H*L* ^1^ could be acidic and thus could be employed for a metallation reaction. Ding and coworkers (Ding *et al.*, 1992 ▸) have reported a series of mercuration reactions on similar Schiff base ligands through electrophilic substitution reactions. On the basis of these observations, we also envisaged that a palladation reaction should take place at the *ortho* position of the tolyl ring in the Schiff base ligand. To investigate this C—H activation step, we attempted to prepare the complex Pd*L* ^1^ *L* ^2^Cl. However, when we treated 2-thio­phene­carboxaldehyde with two equivalents of *p*-toluidine in the presence of Na_2_PdCl_4_ in ethanol solvent at 343 K, none of the expected palladated mol­ecules, Pd*L* ^1^Cl or Pd*L* ^1^ *L* ^2^Cl were observed, and instead we directly isolated the corresponding mono-amine Pd^II^ complex Pd(H*L* ^1^)*L* ^2^Cl_2_,**1**, as red needles in good yield along with a small amount of a yellow solid. The isolated solid was not soluble in common organic solvents. The filtrate of the reaction mixture was allowed to evaporate at room temperature and afforded red needles of a mono-amine Pd^II^ complex. In the FTIR spectra, the C=N stretching frequency in the Pd complex shifts to lower values (1611 cm^−1^) with somewhat weaker intensity in comparison to those of the corresponding free ligand (1615 cm^−1^). Two singlets were also observed at 3777 and 3696 cm^−1^ for the asymmetric and symmetric N—H stretching frequencies, respectively, in the Pd complex. Both frequencies shift to longer wavelengths with weaker intensity in comparison to free *p*-toluidine (3421 and 3338 cm^−1^ for N—H) as a result of the presence of strong N—H⋯Cl hydrogen-bonding inter­actions. This observation was further supported by single-crystal X-ray studies.

Not only does this result contrast with those found for other Schiff base compounds (Dubey *et al.*, 2019 ▸), which readily form a stable palladated complex, but this reaction is also a relatively rare example of a mono-amine Pd^II^ complex. A search of the Cambridge Structural Database (CSD, version 5.43, update of November 2021; Groom *et al.*, 2016 ▸) for structures containing a Pd(NH_2_-phenyl derivative)Cl_2_ fragment gave 51 hits, of which 30 were bis­(amine)PdCl_2_ moieties and among these was the complex Pd(*p*-toluidine)_2_Cl_2_ (YOYWOB; Tay, 2019 ▸) which is relevant for comparison with the title compound. Of the remaining 21, 11 contained the NH_2_ group as part of a chelate ring and only 10 contained a monodentate mono-amine PdCl_2_ complex (BOCTIX, Hadzovic *et al.*, 2008 ▸; HIPDEP, Vicente *et al.*, 1998 ▸; KASNAU, Asma *et al.*, 2005 ▸; OCATEV, OCATIZ, Xia *et al.*, 2021 ▸; OCEPOE, Asma & Kaminsky, 2017 ▸; XEKFEZ, Randell *et al.*, 2006 ▸; XIYLOG, Liu *et al.*, 2002 ▸; XORVIM, Hu *et al.*, 2019 ▸; and YELMOS, Asma *et al.*, 2006 ▸). One of these structures (HIPDEP; Vicente *et al.*, 1998 ▸) is particularly relevant as it contains an *sp^2^* C donor attached to a PdCl_2_(*o*-toluidine) fragment where the major difference with the present structure is the substitution of the *sp^2^* C for *sp^2^* N.

**Figure 1:**
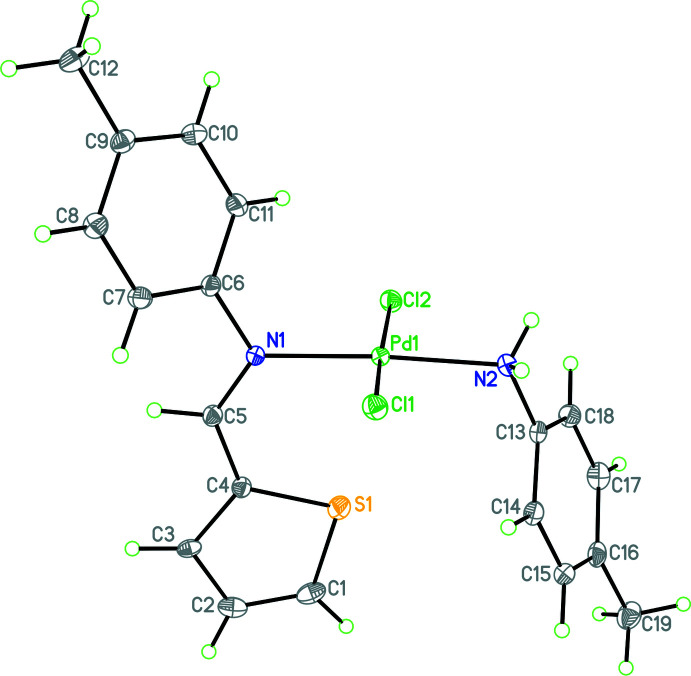
Mol­ecular structure of Pd(H*L* ^1^)*L* ^2^Cl_2_ showing the atom-numbering scheme. Atomic displacement parameters are at the 30% probability level.

An *ORTEP* view of the mol­ecular structure of Pd(H*L* ^1^)*L* ^2^Cl_2_, **1**, is shown in Fig. 1 ▸ and selected bond lengths and bond angles are given in Table 1 ▸. This mono-amine Pd^II^ complex crystallizes in the triclinic space group, *P* 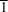 . The primary geometry around the Pd^II^ atom closely resembles square planar (τ_4_′ = 0.069, where 0 = square planar and 1 = tetra­hedral; Okuniewski *et al.*, 2015 ▸). In the (*E*)-1-(thio­phen-2-yl)-*N*-(*p*-tol­yl)methanimine ligand, the phenyl and thio­phene rings are not coplanar because of the steric clash of the hydrogen atoms attached to C5 and C7, exhibiting a dihedral angle of 38.5 (1)°. In addition, the coordination plane (Pd1, Cl1, Cl2, N1, and N2) is almost perpendicular to both the planes of the coordinated *o*-toluidine ring and the C5, C6, N1 fragment [dihedral angles of 84.7 (1) and 72.50 (4)°, respectively]. A search of the CSD (Groom *et al.*, 2016 ▸) for structures containing a Pd(NH_2_-phenyl derivative)Cl_2_ fragment contained 90 observations of the Pd—NH_2_ bond with a mean value of 2.065 (35) Å and minimum and maximum values of 2.028 Å (Baldovino-Pantaleón *et al.*, 2007 ▸) and 2.171 Å (Asma *et al.*, 2005 ▸), respectively. Thus, the length of 2.040 (2) Å in the title compound is slightly shorter than the observed mean value.

A related structure (HIPDEP; Vicente *et al.*, 1998 ▸) contains an *sp^2^* C donor attached to a PdCl_2_(*o*-toluidine) fragment where the major differences with the present structure are the substitution of the *sp^2^* C atom for *sp^2^* N, and the fact that there are *cis* Cl donors, which leads to a substantial *trans* effect involving the Pd—Cl distances. In this structure, the Pd—NH_2_ distance is 2.076 (2) Å. The other related structure (Tay, 2019 ▸) is *trans*-Pd(*o*-toluidine)_2_Cl_2_ in which the Pd—NH_2_ distance is 2.050 (3) Å.

## 3. Supra­molecular features

**Figure 2:**
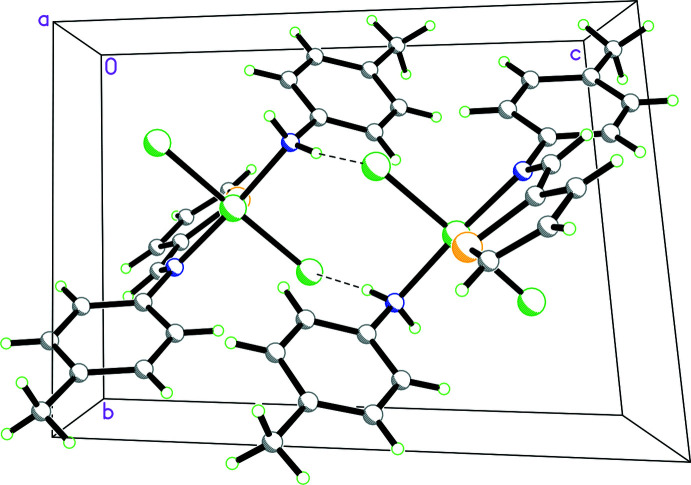
Packing diagram for Pd(H*L* ^1^)*L* ^2^Cl_2_ showing the inter­molecular N—H⋯Cl hydrogen-bonding inter­actions of the *p*-toluidine amine fragment resulting in centrosymmetric dimeric units that are further linked by inter­molecular C—H⋯Cl inter­actions, resulting in chains in the *c*-axis direction where the mean planes of the repeating fragment are oriented in the (110) plane.

The mol­ecules display an inter­esting supra­molecular synthon in the crystal**.** This synthon is based on reciprocal inter­molecular N—H⋯Cl hydrogen-bonding inter­actions (Table 2 ▸) of the *p*-toluidine amine fragment and results in centrosymmetric dimeric units (Fig. 2 ▸). These units are further linked by inter­molecular C—H⋯Cl inter­actions, resulting in chains in the *c*-axis direction where the mean-planes of the repeating fragment are oriented in the (110) plane.

## 4. Synthesis and crystallization

A solution of 2-thio­phene­carboxaldehyde (0.50 ml, 5 mmol) and 2 equivalent of *p*-toluidine (1.07 g, 10 mmol) in 20 ml of freshly distilled ethanol was allowed to stir at room temperature for 1 h. Then Na_2_PdCl_4_ (1.47 g, 10 mmol) was added. The reaction mixture was refluxed under stirring at 343 K for 2 h. A small amount of yellow solid gradually separated during the reaction. After stirring for 3 h the solid was filtered off and the filtrate underwent slow evaporation at room temperature to give red needles of (*p*-CH_3_C_6_H_4_NH_2_)SbHPdCl_2_; yield: 0.80 g, 33%, m.p. 533 K. FT–IR (KBr disk, cm^−1^): 3777 (NH), 3696 (NH), 3406, 2921, 2857, 1611 (CH=N), 1384, 1056, 754. Analysis calculated for C_19_H_20_N_2_Cl_2_PdS: C, 46.98; H, 4.15; N, 5.77. Found: C, 47.10; H, 4.30; N, 6.00%.

## 5. Refinement

Crystal data, data collection and structure refinement details are summarized in Table 3 ▸. All hydrogen atoms were fixed geometrically with their *U*_iso_ values set to 1.2 times that of the phenyl carbons and 1.5 times that of the methyl group. The hydrogen atoms attached to nitro­gen were refined isotropically.

## Supplementary Material

Crystal structure: contains datablock(s) I. DOI: 10.1107/S2056989022004960/tx2049sup1.cif

Structure factors: contains datablock(s) I. DOI: 10.1107/S2056989022004960/tx2049Isup2.hkl

CCDC reference: 2171669

Additional supporting information:  crystallographic information; 3D view; checkCIF report

## Figures and Tables

**Table 1:** Selected geometric parameters (Å, °)

Pd1—N1	2.015 (2)	Pd1—Cl2	2.3082 (7)
Pd1—N2	2.040 (2)	S1—C1	1.702 (3)
Pd1—Cl1	2.3067 (7)	S1—C4	1.721 (3)
			
N1—Pd1—N2	176.02 (9)	N2—Pd1—Cl2	91.00 (7)
N1—Pd1—Cl1	90.55 (6)	Cl1—Pd1—Cl2	174.53 (2)
N2—Pd1—Cl1	86.64 (7)	C1—S1—C4	91.29 (14)
N1—Pd1—Cl2	92.04 (6)		

**Table 2:** Hydrogen-bond geometry (Å, °)

*D*—H⋯*A*	*D*—H	H⋯*A*	*D*⋯*A*	*D*—H⋯*A*
N2—H2*N*2⋯Cl2^i^	0.85 (2)	2.45 (2)	3.273 (2)	163 (2)
C2—H2*A*⋯Cl1^ii^	0.95	2.97	3.779 (3)	144
C5—H5*A*⋯Cl1^iii^	0.95	2.98	3.759 (3)	140
C7—H7*A*⋯Cl1^iii^	0.95	2.80	3.629 (3)	147
C11—H11*A*⋯Cl2	0.95	2.96	3.711 (3)	137

Symmetry codes: (i) 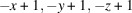 ; (ii) 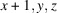 ; (iii) 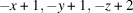 .

**Table 3:** Experimental details

Crystal data	
Chemical formula	[PdCl_2_(C_7_H_9_N)(C_12_H_11_NS)]
*M*_r_	485.73
Crystal system, space group	Triclinic, *P* 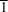
Temperature (K)	100
*a*, *b*, *c* (Å)	9.2135 (4), 9.4060 (4), 12.9032 (5)
α, β, γ (°)	79.866 (2), 70.000 (2), 67.753 (2)
*V* (Å^3^)	971.17 (7)
*Z*	2
Radiation type	Mo *K*α
μ (mm^−1^)	1.34
Crystal size (mm)	0.21 × 0.16 × 0.10

Data collection	
Diffractometer	Bruker APEXII CCD
Absorption correction	Multi-scan (*SADABS*; Krause *et al.*, 2015 ▸)
*T*_min_, *T*_max_	0.622, 0.747
No. of measured, independent and observed [*I* > 2σ(*I*)] reflections	22977, 7379, 5166
*R*_int_	0.074
(sin θ/λ)_max_ (Å^−1^)	0.771

Refinement	
*R*[*F* ^2^ > 2σ(*F* ^2^)], *wR*(*F* ^2^), *S*	0.045, 0.090, 1.04
No. of reflections	7379
No. of parameters	236
No. of restraints	3
H-atom treatment	H atoms treated by a mixture of independent and constrained refinement
Δρ_max_, Δρ_min_ (e Å^−3^)	0.75, −1.05

Computer programs: *APEX2* (Bruker, 2005 ▸), *SAINT* (Bruker, 2002 ▸), *SHELXT* (Sheldrick, 2015*a* ▸), *SHELXL2018/3* (Sheldrick, 2015*b* ▸) and *SHELXTL* (Sheldrick, 2008 ▸).

## supplementary crystallographic information

### Crystal data

**Table:**

[PdCl_2_(C_7_H_9_N)(C_12_H_11_NS)]	*Z* = 2
*M**_r_* = 485.73	*F*(000) = 488
Triclinic, *P*1	*D*_x_ = 1.661 Mg m^−^^3^
*a* = 9.2135 (4) Å	Mo *K*α radiation, λ = 0.71073 Å
*b* = 9.4060 (4) Å	Cell parameters from 7754 reflections
*c* = 12.9032 (5) Å	θ = 2.6–31.6°
α = 79.866 (2)°	µ = 1.34 mm^−^^1^
β = 70.000 (2)°	*T* = 100 K
γ = 67.753 (2)°	Prism, red-orange
*V* = 971.17 (7) Å^3^	0.21 × 0.16 × 0.10 mm

### Data collection

**Table:**

Bruker APEXII CCD diffractometer	5166 reflections with *I* > 2σ(*I*)
φ and ω scans	*R*_int_ = 0.074
Absorption correction: multi-scan (SADABS; Krause *et al.*, 2015)	θ_max_ = 33.2°, θ_min_ = 2.6°
*T*_min_ = 0.622, *T*_max_ = 0.747	*h* = −14→14
22977 measured reflections	*k* = −14→14
7379 independent reflections	*l* = −19→19

### Refinement

**Table:**

Refinement on *F*^2^	Primary atom site location: structure-invariant direct methods
Least-squares matrix: full	Secondary atom site location: difference Fourier map
*R*[*F*^2^ > 2σ(*F*^2^)] = 0.045	Hydrogen site location: mixed
*wR*(*F*^2^) = 0.090	H atoms treated by a mixture of independent and constrained refinement
*S* = 1.04	*w* = 1/[σ^2^(*F*_o_^2^) + (0.0211*P*)^2^ + 0.1526*P*] where *P* = (*F*_o_^2^ + 2*F*_c_^2^)/3
7379 reflections	(Δ/σ)_max_ = 0.001
236 parameters	Δρ_max_ = 0.75 e Å^−^^3^
3 restraints	Δρ_min_ = −1.04 e Å^−^^3^

### Special details

**Table:**

Geometry. All esds (except the esd in the dihedral angle between two l.s. planes) are estimated using the full covariance matrix. The cell esds are taken into account individually in the estimation of esds in distances, angles and torsion angles; correlations between esds in cell parameters are only used when they are defined by crystal symmetry. An approximate (isotropic) treatment of cell esds is used for estimating esds involving l.s. planes.

### Fractional atomic coordinates and isotropic or equivalent isotropic displacement parameters (Å^2^)

**Table:**

	*x*	*y*	*z*	*U*_iso_*/*U*_eq_	
Pd1	0.53572 (2)	0.53405 (2)	0.70240 (2)	0.01680 (6)	
Cl1	0.33922 (8)	0.70208 (8)	0.83363 (6)	0.02463 (14)	
Cl2	0.71210 (8)	0.36598 (7)	0.56567 (5)	0.02350 (14)	
S1	0.85995 (9)	0.55990 (9)	0.70536 (6)	0.02886 (16)	
N1	0.6002 (3)	0.3778 (2)	0.82190 (18)	0.0176 (4)	
N2	0.4704 (3)	0.7036 (3)	0.5873 (2)	0.0220 (5)	
H2N1	0.377 (3)	0.770 (3)	0.622 (2)	0.032 (9)*	
H2N2	0.444 (3)	0.674 (3)	0.5400 (19)	0.028 (9)*	
C1	1.0173 (4)	0.5879 (3)	0.7297 (3)	0.0305 (7)	
H1A	1.076419	0.650381	0.682505	0.037*	
C2	1.0496 (3)	0.5118 (3)	0.8234 (3)	0.0298 (7)	
H2A	1.133321	0.516183	0.848589	0.036*	
C3	0.9467 (3)	0.4256 (3)	0.8798 (2)	0.0198 (5)	
H3A	0.952608	0.364962	0.946424	0.024*	
C4	0.8334 (3)	0.4419 (3)	0.8232 (2)	0.0197 (5)	
C5	0.7160 (3)	0.3650 (3)	0.8615 (2)	0.0198 (5)	
H5A	0.724011	0.295542	0.924122	0.024*	
C6	0.4938 (3)	0.2900 (3)	0.8769 (2)	0.0192 (5)	
C7	0.4335 (3)	0.2799 (3)	0.9911 (2)	0.0227 (6)	
H7A	0.463677	0.329462	1.034377	0.027*	
C8	0.3296 (3)	0.1977 (3)	1.0415 (2)	0.0238 (6)	
H8A	0.288941	0.191174	1.119823	0.029*	
C9	0.2829 (3)	0.1243 (3)	0.9811 (2)	0.0225 (6)	
C10	0.3476 (3)	0.1323 (3)	0.8664 (2)	0.0258 (6)	
H10A	0.320210	0.079781	0.823403	0.031*	
C11	0.4510 (3)	0.2156 (3)	0.8141 (2)	0.0240 (6)	
H11A	0.492453	0.221808	0.735826	0.029*	
C12	0.1631 (3)	0.0410 (3)	1.0382 (3)	0.0301 (7)	
H12A	0.190105	−0.017694	1.103815	0.045*	
H12B	0.169958	−0.029332	0.987540	0.045*	
H12C	0.051154	0.115955	1.060209	0.045*	
C13	0.5932 (3)	0.7750 (3)	0.5331 (2)	0.0198 (5)	
C14	0.6077 (3)	0.8804 (3)	0.5877 (2)	0.0223 (6)	
H14A	0.539342	0.903607	0.661152	0.027*	
C15	0.7222 (3)	0.9519 (3)	0.5351 (2)	0.0240 (6)	
H15A	0.731007	1.025059	0.572709	0.029*	
C16	0.8240 (3)	0.9187 (3)	0.4286 (2)	0.0257 (6)	
C17	0.8092 (4)	0.8100 (3)	0.3760 (2)	0.0287 (6)	
H17A	0.878381	0.785164	0.303024	0.034*	
C18	0.6952 (3)	0.7373 (3)	0.4283 (2)	0.0253 (6)	
H18A	0.687823	0.661983	0.391948	0.030*	
C19	0.9476 (4)	0.9977 (4)	0.3690 (3)	0.0348 (7)	
H19A	0.938923	1.073179	0.416289	0.052*	
H19B	0.925215	1.050022	0.300474	0.052*	
H19C	1.058806	0.921028	0.351413	0.052*	

### Atomic displacement parameters (Å^2^)

**Table:**

	*U*^11^	*U*^22^	*U*^33^	*U*^12^	*U*^13^	*U*^23^
Pd1	0.01898 (10)	0.01641 (10)	0.01739 (10)	−0.00721 (7)	−0.00816 (8)	0.00119 (7)
Cl1	0.0253 (3)	0.0249 (3)	0.0226 (3)	−0.0071 (3)	−0.0069 (3)	−0.0032 (3)
Cl2	0.0275 (3)	0.0217 (3)	0.0215 (3)	−0.0066 (3)	−0.0086 (3)	−0.0033 (2)
S1	0.0309 (4)	0.0297 (4)	0.0300 (4)	−0.0159 (3)	−0.0113 (3)	0.0054 (3)
N1	0.0217 (11)	0.0162 (10)	0.0173 (11)	−0.0082 (9)	−0.0082 (9)	0.0017 (8)
N2	0.0245 (12)	0.0213 (12)	0.0232 (13)	−0.0078 (10)	−0.0127 (11)	0.0022 (9)
C1	0.0253 (15)	0.0279 (15)	0.0389 (18)	−0.0150 (12)	−0.0021 (14)	−0.0050 (13)
C2	0.0245 (15)	0.0322 (16)	0.0368 (18)	−0.0118 (12)	−0.0078 (14)	−0.0103 (13)
C3	0.0145 (12)	0.0182 (12)	0.0269 (14)	−0.0071 (10)	−0.0031 (11)	−0.0047 (10)
C4	0.0219 (13)	0.0195 (12)	0.0178 (13)	−0.0081 (10)	−0.0056 (11)	0.0005 (10)
C5	0.0224 (13)	0.0167 (12)	0.0198 (13)	−0.0055 (10)	−0.0075 (11)	−0.0003 (10)
C6	0.0196 (13)	0.0165 (12)	0.0222 (14)	−0.0061 (10)	−0.0089 (11)	0.0022 (10)
C7	0.0232 (14)	0.0246 (14)	0.0227 (14)	−0.0104 (11)	−0.0066 (12)	−0.0026 (11)
C8	0.0229 (14)	0.0250 (14)	0.0215 (14)	−0.0082 (11)	−0.0035 (12)	−0.0024 (11)
C9	0.0189 (13)	0.0171 (12)	0.0296 (15)	−0.0052 (10)	−0.0069 (12)	0.0002 (11)
C10	0.0300 (15)	0.0237 (14)	0.0287 (16)	−0.0128 (12)	−0.0105 (13)	−0.0021 (11)
C11	0.0280 (15)	0.0282 (14)	0.0194 (14)	−0.0127 (12)	−0.0092 (12)	0.0007 (11)
C12	0.0258 (15)	0.0242 (14)	0.0398 (18)	−0.0128 (12)	−0.0059 (14)	0.0010 (13)
C13	0.0224 (13)	0.0169 (12)	0.0200 (13)	−0.0059 (10)	−0.0096 (11)	0.0034 (10)
C14	0.0253 (14)	0.0191 (13)	0.0202 (14)	−0.0061 (11)	−0.0068 (12)	0.0013 (10)
C15	0.0293 (15)	0.0194 (13)	0.0265 (15)	−0.0089 (11)	−0.0127 (13)	0.0011 (11)
C16	0.0211 (13)	0.0239 (14)	0.0301 (16)	−0.0059 (11)	−0.0118 (13)	0.0075 (11)
C17	0.0262 (15)	0.0281 (15)	0.0234 (15)	−0.0030 (12)	−0.0036 (13)	−0.0030 (12)
C18	0.0314 (15)	0.0232 (14)	0.0210 (14)	−0.0081 (12)	−0.0080 (13)	−0.0029 (11)
C19	0.0290 (16)	0.0328 (17)	0.0409 (19)	−0.0140 (13)	−0.0093 (15)	0.0073 (14)

### Geometric parameters (Å, º)

**Table:**

Pd1—N1	2.015 (2)	C8—H8A	0.9500
Pd1—N2	2.040 (2)	C9—C10	1.394 (4)
Pd1—Cl1	2.3067 (7)	C9—C12	1.507 (4)
Pd1—Cl2	2.3082 (7)	C10—C11	1.384 (4)
S1—C1	1.702 (3)	C10—H10A	0.9500
S1—C4	1.721 (3)	C11—H11A	0.9500
N1—C5	1.291 (3)	C12—H12A	0.9800
N1—C6	1.442 (3)	C12—H12B	0.9800
N2—C13	1.446 (3)	C12—H12C	0.9800
N2—H2N1	0.868 (16)	C13—C18	1.372 (4)
N2—H2N2	0.848 (16)	C13—C14	1.382 (4)
C1—C2	1.358 (4)	C14—C15	1.383 (4)
C1—H1A	0.9500	C14—H14A	0.9500
C2—C3	1.410 (4)	C15—C16	1.384 (4)
C2—H2A	0.9500	C15—H15A	0.9500
C3—C4	1.417 (4)	C16—C17	1.392 (4)
C3—H3A	0.9500	C16—C19	1.509 (4)
C4—C5	1.429 (3)	C17—C18	1.386 (4)
C5—H5A	0.9500	C17—H17A	0.9500
C6—C7	1.386 (4)	C18—H18A	0.9500
C6—C11	1.388 (4)	C19—H19A	0.9800
C7—C8	1.376 (4)	C19—H19B	0.9800
C7—H7A	0.9500	C19—H19C	0.9800
C8—C9	1.383 (4)		
			
N1—Pd1—N2	176.02 (9)	C8—C9—C10	118.0 (2)
N1—Pd1—Cl1	90.55 (6)	C8—C9—C12	120.7 (3)
N2—Pd1—Cl1	86.64 (7)	C10—C9—C12	121.3 (3)
N1—Pd1—Cl2	92.04 (6)	C11—C10—C9	121.1 (3)
N2—Pd1—Cl2	91.00 (7)	C11—C10—H10A	119.5
Cl1—Pd1—Cl2	174.53 (2)	C9—C10—H10A	119.5
C1—S1—C4	91.29 (14)	C10—C11—C6	119.6 (3)
C5—N1—C6	118.0 (2)	C10—C11—H11A	120.2
C5—N1—Pd1	124.71 (18)	C6—C11—H11A	120.2
C6—N1—Pd1	116.66 (15)	C9—C12—H12A	109.5
C13—N2—Pd1	113.09 (16)	C9—C12—H12B	109.5
C13—N2—H2N1	112 (2)	H12A—C12—H12B	109.5
Pd1—N2—H2N1	106 (2)	C9—C12—H12C	109.5
C13—N2—H2N2	110 (2)	H12A—C12—H12C	109.5
Pd1—N2—H2N2	113 (2)	H12B—C12—H12C	109.5
H2N1—N2—H2N2	102 (2)	C18—C13—C14	120.4 (3)
C2—C1—S1	113.1 (2)	C18—C13—N2	120.1 (2)
C2—C1—H1A	123.4	C14—C13—N2	119.5 (2)
S1—C1—H1A	123.4	C13—C14—C15	119.7 (3)
C1—C2—C3	113.4 (3)	C13—C14—H14A	120.1
C1—C2—H2A	123.3	C15—C14—H14A	120.1
C3—C2—H2A	123.3	C14—C15—C16	121.1 (3)
C2—C3—C4	110.5 (2)	C14—C15—H15A	119.4
C2—C3—H3A	124.7	C16—C15—H15A	119.4
C4—C3—H3A	124.7	C15—C16—C17	118.1 (3)
C3—C4—C5	122.3 (2)	C15—C16—C19	121.9 (3)
C3—C4—S1	111.66 (19)	C17—C16—C19	120.0 (3)
C5—C4—S1	126.0 (2)	C18—C17—C16	121.2 (3)
N1—C5—C4	128.2 (2)	C18—C17—H17A	119.4
N1—C5—H5A	115.9	C16—C17—H17A	119.4
C4—C5—H5A	115.9	C13—C18—C17	119.5 (3)
C7—C6—C11	119.9 (2)	C13—C18—H18A	120.3
C7—C6—N1	120.8 (2)	C17—C18—H18A	120.3
C11—C6—N1	119.3 (2)	C16—C19—H19A	109.5
C8—C7—C6	119.6 (3)	C16—C19—H19B	109.5
C8—C7—H7A	120.2	H19A—C19—H19B	109.5
C6—C7—H7A	120.2	C16—C19—H19C	109.5
C7—C8—C9	121.8 (3)	H19A—C19—H19C	109.5
C7—C8—H8A	119.1	H19B—C19—H19C	109.5
C9—C8—H8A	119.1		
			
C4—S1—C1—C2	−0.2 (3)	C7—C8—C9—C12	−177.2 (2)
S1—C1—C2—C3	0.4 (3)	C8—C9—C10—C11	−2.2 (4)
C1—C2—C3—C4	−0.4 (3)	C12—C9—C10—C11	176.6 (3)
C2—C3—C4—C5	179.1 (2)	C9—C10—C11—C6	1.2 (4)
C2—C3—C4—S1	0.3 (3)	C7—C6—C11—C10	0.3 (4)
C1—S1—C4—C3	−0.1 (2)	N1—C6—C11—C10	−179.4 (2)
C1—S1—C4—C5	−178.9 (3)	Pd1—N2—C13—C18	102.6 (2)
C6—N1—C5—C4	−178.6 (2)	Pd1—N2—C13—C14	−76.9 (3)
Pd1—N1—C5—C4	−7.9 (4)	C18—C13—C14—C15	2.1 (4)
C3—C4—C5—N1	175.2 (3)	N2—C13—C14—C15	−178.3 (2)
S1—C4—C5—N1	−6.2 (4)	C13—C14—C15—C16	−0.6 (4)
C5—N1—C6—C7	43.1 (3)	C14—C15—C16—C17	−0.6 (4)
Pd1—N1—C6—C7	−128.3 (2)	C14—C15—C16—C19	178.9 (2)
C5—N1—C6—C11	−137.2 (3)	C15—C16—C17—C18	0.3 (4)
Pd1—N1—C6—C11	51.4 (3)	C19—C16—C17—C18	−179.1 (3)
C11—C6—C7—C8	−0.9 (4)	C14—C13—C18—C17	−2.4 (4)
N1—C6—C7—C8	178.8 (2)	N2—C13—C18—C17	178.1 (2)
C6—C7—C8—C9	−0.1 (4)	C16—C17—C18—C13	1.1 (4)
C7—C8—C9—C10	1.6 (4)		

### Hydrogen-bond geometry (Å, º)

**Table:**

*D*—H···*A*	*D*—H	H···*A*	*D*···*A*	*D*—H···*A*
N2—H2*N*2···Cl2^i^	0.85 (2)	2.45 (2)	3.273 (2)	163 (2)
C2—H2*A*···Cl1^ii^	0.95	2.97	3.779 (3)	144
C5—H5*A*···Cl1^iii^	0.95	2.98	3.759 (3)	140
C7—H7*A*···Cl1^iii^	0.95	2.80	3.629 (3)	147
C11—H11*A*···Cl2	0.95	2.96	3.711 (3)	137

Symmetry codes: (i) −*x*+1, −*y*+1, −*z*+1; (ii) *x*+1, *y*, *z*; (iii) −*x*+1, −*y*+1, −*z*+2.
